# Cell Cycle Arrest and Apoptosis in HT-29 Cells Induced by Dichloromethane Fraction From *Toddalia asiatica* (L.) Lam.

**DOI:** 10.3389/fphar.2018.00629

**Published:** 2018-06-12

**Authors:** Xun Li, Zidong Qiu, Qinghao Jin, Guilin Chen, Mingquan Guo

**Affiliations:** ^1^Key Laboratory of Plant Germplasm Enhancement and Specialty Agriculture, Wuhan Botanical Garden, Chinese Academy of Sciences, Wuhan, China; ^2^University of Chinese Academy of Sciences, Beijing, China; ^3^Shanghai Institute of Materia Medica, Chinese Academy of Sciences, Shanghai, China; ^4^Sino-Africa Joint Research Center, Chinese Academy of Sciences, Wuhan, China

**Keywords:** colon cancer, *Toddalia asiatica* Lam., G2/M arrest, apoptosis, reactive oxygen species

## Abstract

The roots of *Toddalia asiatica* (L.) Lam. (TA) has been often used in Chinese folk medicine to treat different diseases, including but not limited to arthritis, injuries, stomachache, and even tumors. However, the anti-cancer effects and the action mechanisms of TA remain elusive. Therefore, we firstly evaluated the effects of different extracts of TA on the growth of human colon cancer cells, and then tried to further elucidate their underlying molecular mechanisms. As a result, the dichloromethane fraction (DF) was found to possess the highest anti-proliferative activity with IC_50_ value at 18 μg/mL among all of the four extracts from TA, and strongly inhibited HT-29 cell growth and halted cell cycle progression in G2/M phase. DF also induced phosphatidylserine externalization and activated caspases -8, -9, and -3, suggesting DF induced apoptosis through intrinsic and extrinsic pathways. Furthermore, we found that HT-29 cell cycle arrest induced by DF could be the result of reactive oxygen species (ROS), as the ROS scavenger *N*-acetyl cysteine (NAC) attenuating it. Taken together, these results indicated that DF induced cell cycle arrest at G2/M phase and apoptosis in HT-29 cells, and could be a promising source for developing natural therapeutics for colon cancer.

## Introduction

*Toddalia asiatica* (L.) Lam. (Rutaceae) (TA) has been widely used as traditional Chinese medicine for the treatment of various diseases in China (Yang et al., 2013; Tong et al., 2014). Modern pharmacologic researches have confirmed that TA extracts have multiple biological activities, including anti-arthritis (Yang et al., 2013), anti-inflammatory (Hao et al., 2004; Balasubramaniam et al., 2011; Kariuki et al., 2013; Tong et al., 2014), anti-microbial (Narod et al., 2004; Duraipandiyan and Ignacimuthu, 2009; Karunai Raj et al., 2012), anti-parasitic (Shan et al., 2014), anti-oxidant (Balasubramaniam et al., 2011; Irudayaraj et al., 2012; Stephen Irudayaraj et al., 2012; Ceballos et al., 2013), anti-platelet (Tsai et al., 1998), anti-malarial (Gakunju et al., 1995; Oketch-Rabah et al., 2000), anti-diabetic (Irudayaraj et al., 2012), anti-tumor (Iwasaki et al., 2006), and analgesic (Hao et al., 2004; Kimang’a et al., 2016). TA extracts are mainly composed of coumarins, alkaloids, benzenoids, and their derivatives (Hu et al., 2014). The pure compounds of TA such as toddaculin, 8-methoxydihydrochelerythrine, 8-methoxynorchelerythrine, ski-mmiamine, benzo[c]phenanthridine derivatives have been shown to inhibit proliferation in diverse types of human cancer cells derived from different tissue origins *in vitro* (Iwasaki et al., 2006, 2010; Vázquez et al., 2012; Hirunwong et al., 2016), suggesting that TA extracts or its bioactive components have a good potential for the discovery and development of novel natural anti-cancer therapeutics. However, the cytotoxicity of *Toddalia asiatica* root extracts and their action mechanisms associated with cell proliferation remained unexplored to date.

On the other hand, according to cancer statistics 2015 in China, a substantial increase in both incidence and mortality of cancer has produced a major public health issue in the country. Among them, colon cancer is ranked the fifth among cancer deaths nationwide (Chen et al., 2016). However, the incidence and mortality of colon cancer in rural areas are higher than that in urban areas, the limited medical resources, such as diagnosis, timely report, and treatment in rural areas were considered to be the most primary factor leading to these results (Chen et al., 2016). In addition, traditional Chinese medicines (TCMs) have played an important part in primary health care in rural areas of China in terms of general availability, considerable curative action, and mild side effects since long time ago, and are becoming an important resource for natural new drug discovery nowadays. More importantly, some natural medicines derived from TCMs are even used in clinic for the treatment of various cancers. In this context, we strived to discover and develop new, affordable, and effective natural therapeutics from TA for the treatment of colon cancers. To this end, we firstly examined the effects of TA extracts on cell cycle development and cell apoptosis, and then tried to explore the potential of TA as a useful natural product against colon cancer.

## Materials and Methods

### Reagents

Sulforhodamine B (SRB), propidium iodide (PI), dimethyl sulfoxide (DMSO), *N*-acetyl cysteine (NAC), ethylenediaminetetraacetic acid (EDTA), phosphatase inhibitor cocktail and the reagents for sodium dodecyl sulfate-polyacrylamide gel electrophoresis (SDS-PAGE) were obtained from Sigma (St. Louis, MO, United States). Fetal bovine serum (FBS) was obtained from Biological Industries Israel Beit Haemek Ltd. (Beit-Haemek, Israel). Dulbecco’s modified Eagle’s medium (DMEM)/high glucose, trypsin, penicillin-streptomycin were acquired from HyClone- GE Healthcare Life Sciences (Logan, UT, United States). The Annexin V-FITC apoptosis Assay kit was obtained from Multi Sciences (Lianke) Biotech Co., Ltd. (Hangzhou, China). Bicinchoninic acid (BCA) assay kit and Laemmli sample buffer were acquired from Beyotime Institute of Biotechnology (Haimen, China). Mouse monoclonal anti-caspase-8 (66093-1-Ig), mouse monoclonal anti-caspase-9 (66169-1-Ig), horseradish peroxidase (HRP)-conjugated GAPDH antibody (HRP-60004), HRP-conjugated secondary antibodies (SA00001-1), polyvinylidene fluoride (PVDF) membranes, radio-Immunoprecipitation assay (RIPA) buffer, and enhanced chemiluminescence (ECL) assay kit were purchased from Proteintech Group (Chicago, IL, United States). ROS-sensitive fluorescent dye 2O, 7O-dichlorfluorescein-diacetate (H_2_DCF-DA) was purchased from Molecular Probes (Eugene, OR, United States). All other chemicals and reagents with the highest quality were obtained from Sigma (St. Louis, MO, United States).

### Preparation of Plant Extracts

**FIGURE 1:**
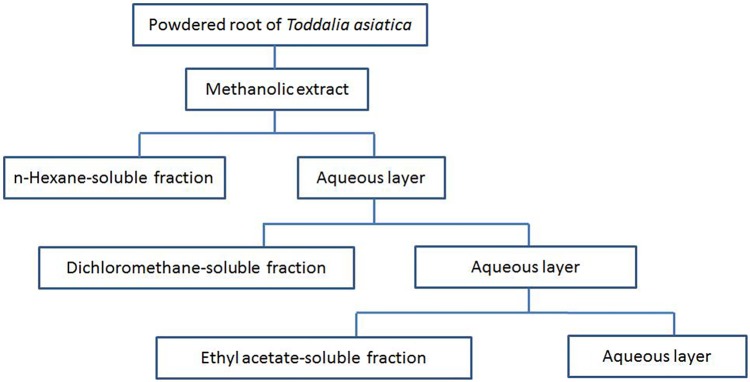
Partitioning scheme of the extracts from root of *Toddalia asiatica* Lam. (TAR)

Roots of TA were collected from Enshi Tujia and Miao Autonomous Prefecture located in western Hubei Province China in July 2016, and authenticated by the botanist (Prof Guangwan Hu) at Wuhan Botanical Garden, Chinese Academy of Sciences, where a voucher specimen (No. TA-20160715-001) was deposited. The collected roots were dried and stored at room temperature. Then, the dried roots were ground into powder by an electric mill. The powders of the roots were extracted with methanol (MeOH) at room temperature three times with each time for 7 days. The whole extracts were combined and filtered, and then the resultant solution was evaporated under vacuum to obtain a crude extract. Fractionation procedures were based on previously described methods (Parsaee et al., 2013). In brief, the resultant solution was successively partitioned with n-hexane, dichloromethane (CH_2_Cl_2_), and ethyl acetate (EtOAc). The n-Hexane, dichloromethane, and ethyl acetate fractions were evaporated under vacuum to afford dried residues for each fraction. These dried fractions were reserved at 4°C before subsequent analysis. The partitioning strategy of the TA roots (TAR) is showed in **Figure 1**. Stock solutions of the extracts at a final concentration of 200 mg/mL were prepared by dissolving them in DMSO before use in further experiments.

### Cell Culture

The human colon cancer cell lines, such as HT-29 (HTB-38), SW480 (CRL-228), LoVo (CRL-229), and HCT-116 (CCL-247) were purchased from the American Type Culture Collection (ATCC, Manassas, VA, United States) and cultured in DMEM/high glucose containing 10% FBS, 1% penicillin-streptomycin. These cells were maintained at 37°C in a cell incubator with 95% air and 5% CO_2_. The culture medium was refreshed two or three times a week.

### Cell Proliferation Assay

The anti-proliferation potential of extracts of TAR was examined using Sulforhodamine B (SRB) growth assay (Skehan et al., 1990). Approximately 10^4^ cells were seeded into 96-well plates, and then positioned in the cell incubator overnight. The cells were subsequently treated with or without various concentrations of different extracts of TAR. The final concentration of DMSO was less than 0.2% (v/v) in all experiments. Cells treated with the same amount of DMSO were served as controls. The exposed cells were then incubated in cell incubator for 72 h. After 72-h treatment, the culture medium was aspirated, and the cells treated with extracts were then fixed using 10% (w/v) trichloroacetic acid (TCA) at 4°C for 1 h. The fixed cells were washed four times with distilled-deionized H_2_O to get rid of TCA and dried in air at room temperature. Then, 0.4% (w/v) SRB in 1% acetic acid was added in each well for 1 h at room temperature. The unbound dye was quickly rinsed three times using 1% acetic acid and then dried at room temperature. The bound dye in each well was solubilized with 100 μL of 10 mM Tris base solution (pH 10.5) in a gyratory shaker for 20 min. the absorbance (OD) of each well was measured at 565 nm using a microplate reader (M200 PRO, TECAN). The equation below was used to calculate the % cell growth inhibition: the % Cell growth inhibition = (100 × (mean OD_control_ - mean OD_sample_))/mean OD_control_.

The IC_50_ values were determined using non-linear regression analysis with SigmaPlot 12.0.

### Cell Cycle Assay

Cells were seeded in 12-well plates. After overnight culture, the cells were treated with or without TAR extracts at different concentrations for 24 h, and vehicle treatment was served as control group. After treatments, both floating and attached cells were collected by trypsinization, and washed twice with PBS (phosphate-buffered saline) before the fixation in 2 mL cold 70% (v/v) ethanol at -20 °C overnight. Centrifugation was then used to remove the fixative solution. After washing with PBS once again, the fixed cells were re-suspended in PI staining solution (PBS, 0.2 mg/mL RNase A, 20 μg/mL PI and 0.1% Triton X-100 added extemporaneously), and then placed in the dark for more than 2 h at 4°C. Usually, 10^4^ events were acquired for each treatment, and the cell cycle profile was analyzed with BD FACSVerse (Becton Dickinson), while the cell cycling distributions were determined using ModFit LT software (Becton Dickinson). All of the experiments above were done in triplicate, and the results were expressed as the percentage of cells in a particular phase.

### Annexin V-FITC/PI Double Staining Assay

The seeding cells in 12-well plates were incubated overnight, the medium was changed and the TAR extracts of interest were added. The floating and adherent cells were collected by trypsinization after 24 h treatment with the extracts. The collected cells were washed three times using PBS, and then stained with Annexin V-FITC and PI according to the instruction manual elsewhere. Samples were acquired on a BD FACSAria III Cell Sorting System (Becton Dickinson) before analysis using the BD FACSDiva software 6.1.3 (Becton Dickinson). Early apoptosis was designated as annexin positive/PI negative and late apoptosis was designated as annexin positive/PI positive, whereas necrosis was defined as annexin negative/PI positive.

### Western Blotting

Cells were treated with or without dichloromethane fraction (DF) of TAR extracts at the indicated concentrations and were harvested. The collected cells were lysed in RIPA buffer containing protease and phosphatase inhibitor cocktail on ice for 40 min, and then centrifuged at 12,000 rpm for 10 min at 4°C. The total protein concentrations in each sample were measured with a BCA protein assay kit. For western blot analysis, the protein lysates were denatured in Laemmli sample buffer. The same amounts of the protein samples were electrophoresed on SDS-PAGE before transfer onto PVDF. After blocked with blocking buffer (5% non-fat milk, 20 mM Tris–HCl, 500 mM NaCl, and 0.1% Tween 20) at room temperature for 1 h, the PVDF membranes were incubated with corresponding primary antibodies at 4°C overnight. After extensive washing with buffer (20 mM Tris–HCl, 500 mM NaCl, and 0.1% Tween 20), the membranes were incubated for 1 h at room temperature with horseradish-peroxidase-conjugated secondary antibodies. The protein-antibody complexes were detected by ECL detection Kit. The signal was visualized using the ECL chemiluminescence system (GE Healthcare). In order to verify the same protein loading, the blots were reprobed with anti-GAPDH antibody in all of the western blot experiments.

### Measurement of Intracellular Reactive Oxygen Species (ROS)

Intracellular reactive oxygen species (ROS) production was measured using the ROS indicator, H_2_DCF-DA. Briefly, cells were seeded in 12-well plates. After overnight culture, the cells were treated with or without DF at different concentrations for 24 h. After treatments, the treated cells were detached with trypsin–EDTA, and washed twice with PBS. Cells were immediately resuspended in 0.2 mL PBS containing H_2_DCF-DA at the final concentration of 10 μM, and reacted in the dark at 37°C for 30 min, and washed with PBS to remove free dyes. ROS production of cells was then evaluated by flow cytometry (BD FACSAria III). Not less than 10^4^ cells from each treatment were analyzed. FlowJo 7.6 (Tree Star) and SigmaPlot 12.0 were used to analyze data.

### Statistical Analyses

The significance of the differences in measure of variables between treatment groups and controls was performed using one-way ANOVA or paired *t*-test followed by Dunnett’s or Bonferroni’s multiple comparison tests. Statistical analysis was carried out using SPSS 13.0. For all experiments, *P* value of ≤0.05 was considered as statistically significant.

## Results

### Effects of TAC Extracts on Cell Growth of HT-29 Cells

The crude methanol extracts and several other fractions of TAR were examined for their anti-proliferative activity with HT-29 cells. Among all the fractions tested, DF, showed the highest anti-proliferative effects with the IC_50_ value at 18 μg/mL (**Table 1**), suggesting that DF significantly inhibited the growth of HT-29 cells. Thus, DF was selected for further studies. However, the action mechanisms of DF have not been explored.

**Table 1:** IC_50_ values (μg/mL) of different fractions of TAR against HT-29 cells.

Cell line	Fraction				
	
	MeOH	n-hexan	CH_2_Cl_2_	EtOAc	water
HT-29	>150	>200	18	131	>200


### DF Caused Cell Cycle Arrest at the G2/M Phase

**FIGURE 2:**
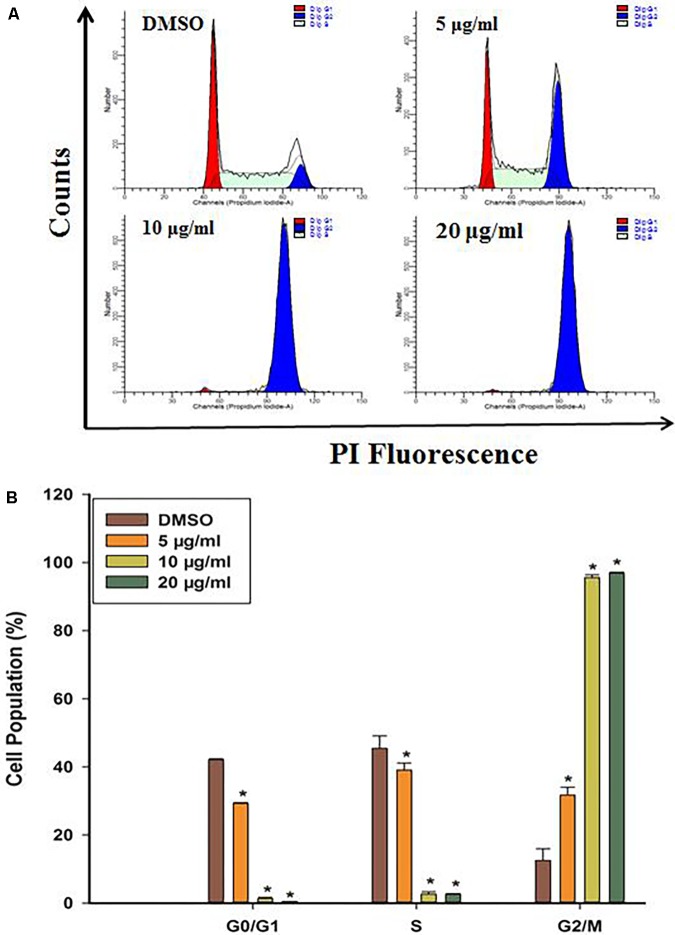
DF induced cell cycle arrest at the G2/M phase in HT-29 cells treated with DMSO (control) or various concentrations of DF for 24 h and stained with PI. Cells were then subjected to cell cycle analysis using BD FACSVerse. **(A)** Representative profiles of cell cycle distribution in HT-29 cells after 24 h of treatment with DMSO or varying concentrations of DF. **(B)** The percentages of cell populations in the G0/G1, S, and G2/M phases. The data were shown as means ± SD from at least three independent experiments. ^∗^*p* < 0.05 compared with the control.

To probe whether cell cycle arrest contributed to cell growth suppression by DF, we firstly evaluated how DF would affect cell cycle distribution in HT-29 cells by PI staining, and the DNA content was examined by flow cytometry. As shown in **Figure 2**, increasing concentration of DF caused a significant increase in cell population at the G2/M phase along with decrease in cell population in G0/G1 and S phase cells. These results showed that DF inhibited the growth of HT-29 cells with cell cycle arrest at G2/M phase.

Due to the discrepancies in molecular features of many colon cancer cell lines (Ahmed et al., 2013), questions were raised whether DF caused the similar effects on other colon cancer. We then examined the effects of DF treatment on several other colon cancer cells, such as HCT-116, SW480, and LoVo cells. Similar to HT-29 cells, DF also inhibited the growth of HCT-116, SW480, and LoVo cells with cell cycle arrest at G2/M phase (as shown in Supplementary Figures S1, S2). These results suggested that DF caused cell cycle arrest at G2/M phase is not specific for HT-29 cells, and may represent a general action mechanism against other colon cancer cells.

### DF Induced Phosphatidylserine Externalization in HT-29 Cells

**FIGURE 3:**
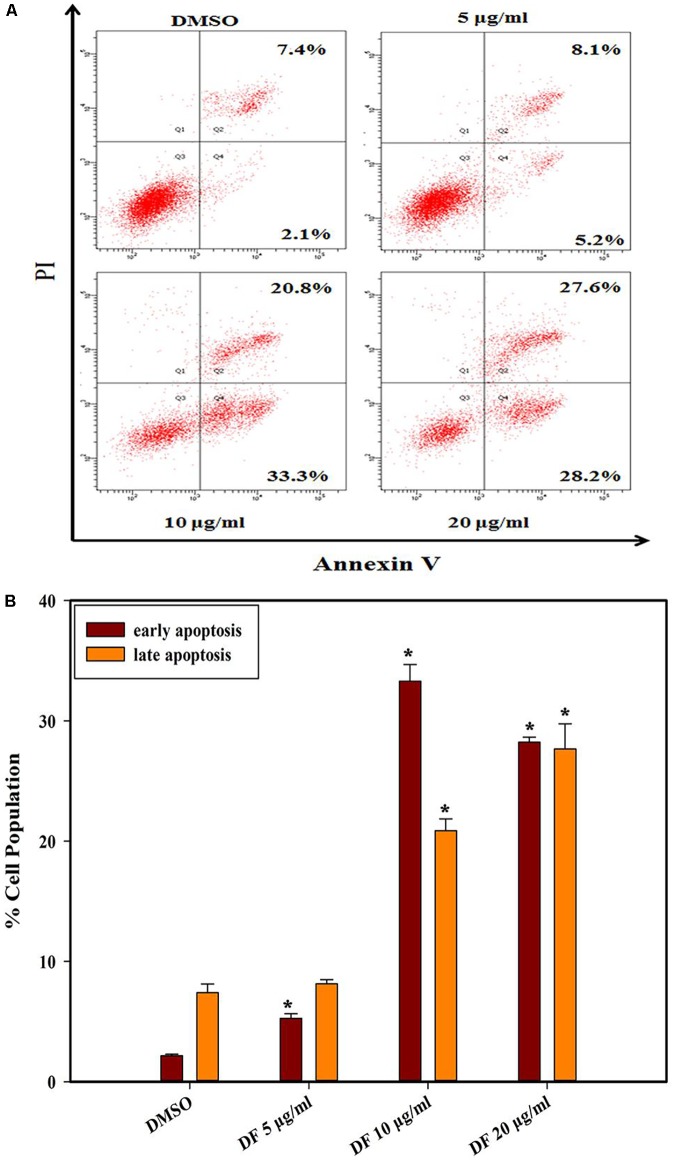
The effects of DF on the externalization of PS in HT-29 cells after 24 h. **(A)** The different distribution of HT-29 cells stained with annexin V-FITC/PI in a dual parametric dot plots of PI fluorescence (*Y*-axis) versus annexin V-FITC fluorescence (*X*-axis). **(B)** Bar chart indicates the proportion of early apoptosis and late apoptosis as compared to DMSO-treated cells. Values are expressed as mean ± SD from at least three independent experiments. The asterisk indicated significantly different values from DMSO-treated control cells (^∗^*P* ≤ 0.05).

Phosphatidylserine (PS) externalization is a typical characteristic of early phase apoptosis (Fadok et al., 1998). In this context, we further explored whether anti-proliferative effects of DF on HT-29 cells were related to apoptosis using the Annexin V-FITC/PI double staining. As illustrated in **Figure 3**, the HT-29 cells underwent dose-dependent apoptosis after 24 h treatment with DF. Moreover, DF also induced apoptosis in other colon cancer cells (Supplementary Figure S3). These data suggested that DF-induced suppression of cell growth could be a result of cell apoptosis.

### DF Induced Caspase-8 and -9 Activation in HT-29 Cells

**FIGURE 4:**
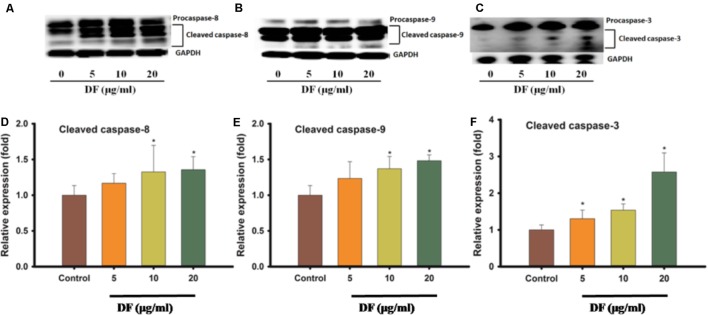
The effects of DF on the activation of caspases -8, -9, and -3. The HT-29 cells were treated with DF for 12 h and analyzed by Western blotting, and equal loading amount was measured by probing for GAPDH, **(A)** caspases -8; **(B)** caspases -9; **(C)** caspases -3. Quantitation of protein levels of cleaved caspases protein levels in HT-29 cells treated with DF for 12 h. **(D)** Cleaved caspases -8; **(E)** Cleaved caspases -9; **(F)** Cleaved caspases -3. The asterisk indicated significantly different values from DMSO-treated control cells (^∗^*P* ≤ 0.05).

Because apoptotic cell death can happen via many signal pathways, we set out to probe which pathway of apoptosis was elicited by DF treatment. The activation of some initiator caspases, like caspase-8 (extrinsic pathway) and caspase-9 (intrinsic pathway), and the activation of executor caspase-3 (a downstream effector of both pathways) was analyzed using western blotting. As shown in **Figure 4**, DF treatment of HT-29 cells resulted in a dose-dependent increase in the active forms of caspase-8 and -9 after 12 h. Also, an increase in procaspase-3 active form was observed in the HT-29 cells. These findings suggested that DF-induced apoptosis is associated with both intrinsic and extrinsic pathways.

### The Accumulation of ROS Induced by DF Participated in the Cell Cycle Arrest of HT-29 Cells

**FIGURE 5:**
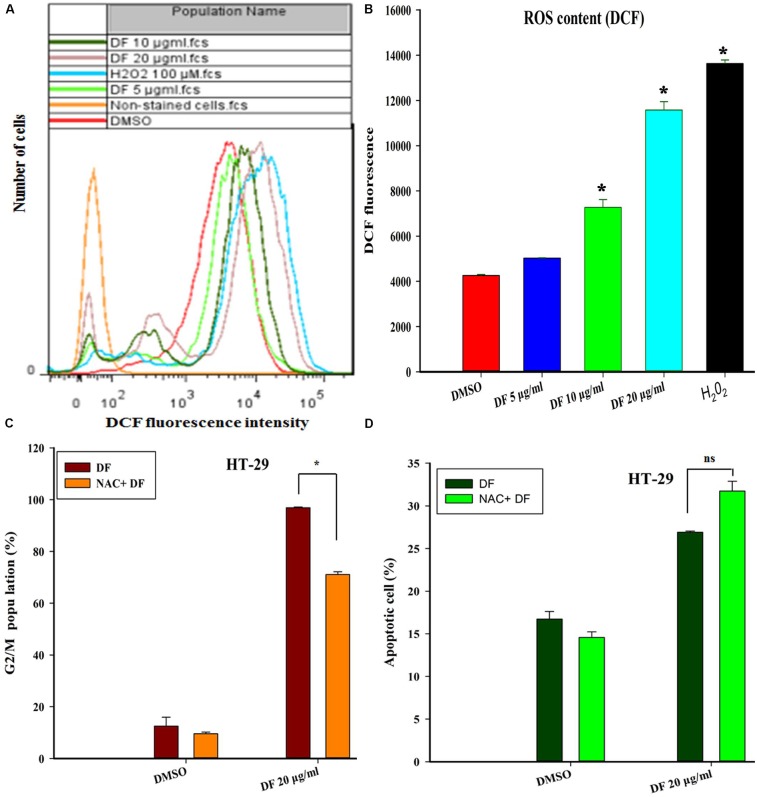
DF induced the elevation of ROS, which contributed to HT-29 cells’ G2/M phase arrest, but not the cell apoptosis. **(A)** HT-29 cells were treated with DMSO (control) and various concentrations of DF for 24 h, and then loaded with H_2_DCF-DA. The mean DCF fluorescence was measured by flow cytometry. 100 μM H_2_O_2_ was used as positive control; **(B)** histograms are representative of mean DCF fluorescence of three independent experiments; ^∗^*P* ≤ 0.05; **(C)** the cells were incubated for 2 h in the presence or absence of NAC (20 mM), and DF (20 μg/mL) was added and incubated for 24 h. Then, the cell cycle distribution was analyzed using flow cytometry. The percentage of cells in each population is shown as the mean ± SD from three independent experiments. Here, ns represents not significant. **(D)** The cells were pre-incubated for 2 h in the presence or absence of NAC (20 mM), and then DF (20 μg/mL) was added and incubated for 24 h. The induction of apoptosis was determined by flow cytometry. Statistical analysis of the percentages of the apoptotic cells was performed, and data shown are from three independent experiments, ^∗^*P* ≤ 0.05.

Numerous previous studies have shown that ROS generation plays a vital role in cell cycle progression and apoptosis in various cancer cells treated with various types of anticancer agents (Sauer et al., 2001; Fleury et al., 2002). Thus, we investigated whether DF-induced cell cycle arrest and apoptosis was directly associated with ROS formation in HT-29 cells. In this study, the intracellular ROS level was measured using the ROS-sensitive dye H_2_DCF-DA. **Figures 5A,B** show that treatment of HT-29 cells with DF at 10 μg/mL and 20 μg/mL for 24 h resulted in a dramatic increase in the mean DCF fluorescence, indicating that DF could induce intracellular ROS generation. Next, to ascertain whether DF-induced cell cycle arrest or apoptosis resulted from too much production of ROS, pre-treatment of HT-29 cells with the antioxidant (NAC) for 2 h was conducted before supplementing with DF for a further 24-h treatment. Strikingly, NAC blocked the DF-induced increase in the G2/M proportion (**Figure 5C**). However, DF-induced increase of cell apoptosis was not prevented by the same treatment (**Figure 5D**). Taken together, these findings indicated that DF-induced cell cycle arrest was, at least to a certain degree, associated with an ROS-dependent cell cycle progression. However, the induction of apoptosis did not involve ROS.

## Discussion

TCMs are viewed as “the great treasure house of mankind”. Many valuable “treasures” in TCMs are gradually discovered by virtue of modern science and technology development. Many TCMs or their constituents have served as potential candidates for anticancer drugs or already been used in clinical trials, such as quercetin, genistein, catechins, and anthocyanidins (Khan et al., 2008; Korkina et al., 2009). TCMs have a long history to battle cancer in China. There is a new trend in discovering and developing novel and affordable anti-cancer drugs from TCMs. In the present study, we decided to search for new natural compounds with an ability to affect HT-29 cells proliferation and explored the likely underlying molecular mechanisms. To this end, DF of TA showed potential anti-cancer activity against HT-29 human colon cancer cells, and was attributable to its effective suppression of cell cycle progression, and induction of apoptosis via intrinsic (mitochondrial) and extrinsic (death receptor) pathways. These results may lay a foundation for the development of novel anti-colon cancer drugs with affordable costs and little or no side effects.

It is well known that cell cycle progression governs cell proliferation (Dickson and Schwartz, 2009), and its malfunction is one of the crucial stages in the development of cancer (Williams and Stoeber, 2012). Thus, the control over cell cycle progression by activation of cell cycle arrest might be an appropriate approach for cancer therapy (Carnero, 2002; Wang et al., 2009). Cell cycle arrest in response to stimulus is indispensable for repairing cellular damage, and the control machineries for hampering cell cycle transition after cellular damage are called by cell cycle checkpoints (Flatt and Pietenpol, 2000). Flow cytometric analysis revealed a significant accumulation of cells in the G2/M phase, along with decreases in the percentage of cells in the G0/G1 and S phase in comparison to the control cells (**Figure 2**). These results implied that DF suppressed cell proliferation through cell cycle arrest at the G2/M phase in a dose dependent fashion, and the G2/M checkpoint prevented the entry of DNA lesion cells into M-phase, providing a chance to repair and discontinue proliferation of damaged cells (Stark and Taylor, 2006). It has been reported that cyclins and CDKs (cyclin-dependent kinases) are essential for the cells to move into the different phases of cell cycle, and the CDC2 (CDK1)-Cyclin-B kinase activity plays a vital role in controlling the transition from G2 phase to the M phase. We thus conjectured that DF might affect CDC2 (CDK1)-Cyclin-B kinase activity of HT-29 cells, and resulted in distinct cell cycle perturbation.

In another aspect, wild-type *p53* is critically involved in the regulation of cell cycle arrest (Vousden, 2000; Woods and Vousden, 2001), and *p53* gene mutation or deletion, and loss of *p53* normal function could cause various types of human tumors. In the present study, we have explored whether or not the influence of DF on the cell cycle is *p53* dependent by comparing responses in two cell lines with mutant p53 (HT-29 and SW480) with two cell lines with wild-type (wt) p53 (LoVo and HCT-116), and found the same trends in cell cycle arrest caused by DF (Supplementary Figure S2). These results indicated that *p53* status was not directly linked to the responses observed in cell cycle following treatment with DF.

Furthermore, apoptosis plays a crucial role in governing tumorigenesis and treatment responses, and current treatment strategies for most cancer, including chemotherapy, radiation therapy, and surgery, often involve triggering cellular apoptosis signaling pathways of cancer cells (Ghobrial et al., 2005; Fulda and Debatin, 2006; Lee et al., 2015). In addition, caspases, closely linked to apoptosis, are produced as inactive zymogens in most cells. Active initiator caspases (caspase -8, -9) can often activate other downstream caspases termed executioner caspases (caspase -3, -6, -7), thus causing caspase cascade, which strengthens the apoptotic signaling pathway resulting in cell death (Degterev et al., 2003). In the present study, we found that DF induced apoptosis in HT-29 cells through the induction of phosphatidylserine externalization and the activation of initiator and effector caspases (caspase -8, -9, -3) (**Figures 3**, **4**). These data showed that both the extrinsic and intrinsic pathways were responsible for DF-induced cell apoptosis. In the future, caspase inhibitors could be used to substantiate the activation of caspases involved in apoptosis signaling.

In addition, the Bcl-2 family proteins, like Bax are also critical factors in regulating cellular apoptosis processes (Green and Reed, 1998; Wei et al., 2001; Choi and Singh, 2005), and Bax usually locates in the cytoplasm, however, under certain circumstances, such as when the cell is under stress, the Bax translocates to the mitochondria, leading to apoptosis. To explore the role of the *Bax* in apoptosis caused by DF, we investigated and compared the induction of apoptosis in two cell lines with wild-type (wt) *Bax* (HT-29 and SW480) and two cell lines with mutant *Bax* (LoVo and HCT-116) with or without DF treatment. As a result, phosphatidylserine externalization was observed in cell lines with wt *Bax* and mutant *Bax* (Supplementary Figure S3), suggesting that DF-induced apoptosis did not depend on Bax.

Last but not least, ROS is regarded as an important messenger molecule to regulate cell death and survival in cancer cells (Sauer et al., 2001; Raza et al., 2017). Under normal physiological conditions, physiological levels of ROS stimulates cell proliferation and survival, whereas elevated levels of ROS can damage many cellular constituents, such as lipids, protein and DNA, resulting in cell cycle arrest and apoptosis (Boonstra and Post, 2004; Maiuri et al., 2007). Changes in ROS concentrations play a vital role in tumorigenesis, and are considered as the hopeful therapeutic approaches in cancer therapy (Pelicano et al., 2004; Trachootham et al., 2009; Raza et al., 2017). In this work, it was found DF induced ROS generation in a concentration-dependent manner in HT-29 cells. Furthermore, pretreatment with ROS scavenger NAC could reverse DF-induced cell cycle arrest and did not prevent DF-induced cell apoptosis (**Figure 5**), which indicated that ROS generation was at least partially required for cell cycle progression, and the induction of apoptosis did not directly associate with ROS.

In the past decades, a large number of compounds have been isolated from TA including alkaloids, coumarins, benzenoids, etc. (Hu et al., 2014). The cytotoxicity of some of these compounds have been reported (Iwasaki et al., 2006, 2010; Vázquez et al., 2012; Hirunwong et al., 2016); toddaculin at 250 μM was able to induce apoptosis in U-937 cells though decreased phosphorylation levels of ERK (extracellular signal-regulated protein kinase) and Akt (protein kinase B) (Vázquez et al., 2012). Skimmiamine could inhibit the growth of MCF-7 cells *in vitro* with an IC_50_ value of 8.7 mg/mL; 8-methoxydihydrochelerythrine displayed an inhibitory effect on the growth of KB, NCI-H187, MCF-7, and Vero cells lines with IC_50_ values ranging from 0.8 to 11.6 mg/mL (Hirunwong et al., 2016). Dihydronitidine showed cytotoxicity against A549, COLO-201, MIA-PaCa2, A431, KATOIII, SKBR-3 cells with IC_50_ values ranging from 0.19 to 4.60 mg/mL and was inactive against normal cells lines (Iwasaki et al., 2006). In addition, Benzo[c]phenanthridine and secobenzo[c]phenantridine alkaloids exhibited considerable antiproliferation activity against various types of tumor cells (Iwasaki et al., 2010; Hu et al., 2014). Therefore, the presence of these compounds, especially prenylated coumarins and benzophenanthridine alkaloids may explain the inhibition of cell proliferation of HT-29 cells by DF in this study but further studies will be needed in order to verify this speculation.

## Conclusion

In order to explore the potential and action mechanisms of TA as a useful folk medicine against colon cancer, our study firstly revealed the DF fraction of TAR exhibited the higher anti-proliferative effects than the other four fractions after compared their IC_50_ values against HT-29 cells. Based on this finding, we further explored the anti-proliferative effects of DF on HT-29 cells *in vitro* and the potential action mechanisms of DF at the molecular level. We found that DF dramatically halted cell cycle progression at G2/M phase and induced cell apoptosis via intrinsic and extrinsic pathways, as evidenced by the activation of the initiator caspase-8 and caspase-9, and the activation of executor caspase-3. However, DF-induced apoptosis did not depend on Bax. In addition, *p53* status was not directly linked to the response observed in cell cycle arrest upon DF treatment, while ROS generation was at least partially required for cell cycle progression. Not only may these novel findings lay foundation for more interests in future study on this valuable medicinal plant, but also could open a new avenue for further exploration of TAR as a new natural medicine for battling colon cancer. Collectively, DF has great potential to be explored as a promising natural source for the treatment of colon cancer in the near future.

## Author Contributions

MG and XL conceived and designed the study. XL, ZQ, QJ, and GC performed the experiments, analyzed the data, and wrote the manuscript. All authors approved and reviewed the final manuscript.

## Conflict of Interest Statement

The authors declare that the research was conducted in the absence of any commercial or financial relationships that could be construed as a potential conflict of interest.
