# Supplementary material for: Cell Cycle Arrest and Apoptosis in HT-29 Cells Induced by Dichloromethane Fraction From Toddalia asiatica (L.) Lam
Source: Front Pharmacol. 2018 Jun 12;9:629. doi: 10.3389/fphar.2018.00629 (PMC6008524; doi:10.3389/fphar.2018.00629)
Supplement: Supplementary file 1 [file Data_Sheet_1.docx]

Supplementary Material

Induction of cell cycle arrest and apoptosis in HT-29 human colon cancer cells by dichloromethane fraction from *Toddalia asiatica*

**Xun Li ^1, 2^, Zidong Qiu^1, 2^, Qinghao Jin ^1, 3^, Guilin Chen^1, 4^, Mingquan Guo^1, 4^ ***

*** Correspondence:** Corresponding Author: [guomq@wbgcas.cn](mailto:guomq@wbgcas.cn)


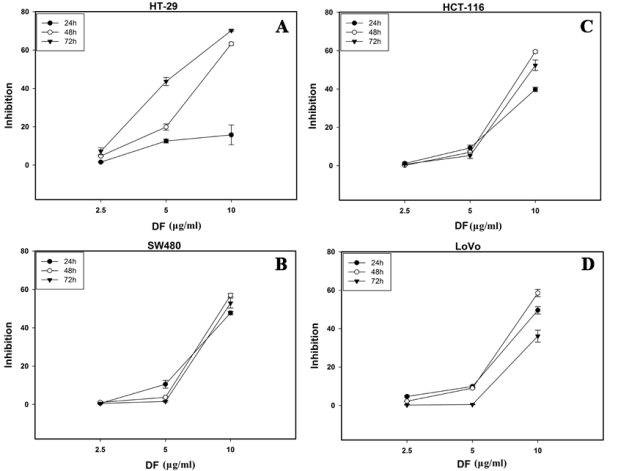


**Supplementary Figure 1** | DF inhibited the growth of the human colon cancer cell lines, HT-29 cells, SW480 cells, HCT-116 cells, LoVo cells. Cells treated with various concentrations of DF for 24, 48, and 72h. SRB assay was used to assess colon cancer cell proliferation. (A) HT-29 cells, (B) SW480 cells, (C) HCT-116 cells, (D) LoVo cells.


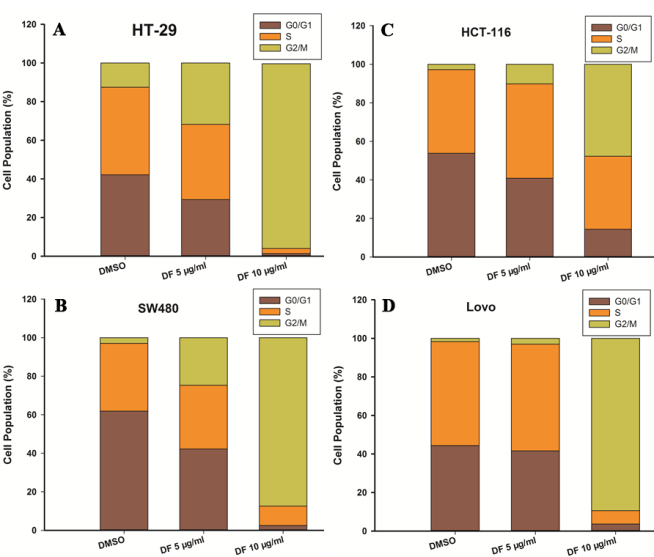


**Supplementary Figure 2 |** DF induced G2/M cell cycle arrest in the human colon cancer cell lines, HT-29 cells, SW480 cells, HCT-116 cells, LoVo cells. Cells were treated with DMSO (control) and DF (5 and 10 μg/ml) for 24 h. The distribution of cell cycle was assessed by flow cytometry. The percentage of cells in each phase is showed as mean from three independent experiments. (A) HT-29 cells, (B) SW480 cells, (C) HCT-116 cells, (D) LoVo cells.


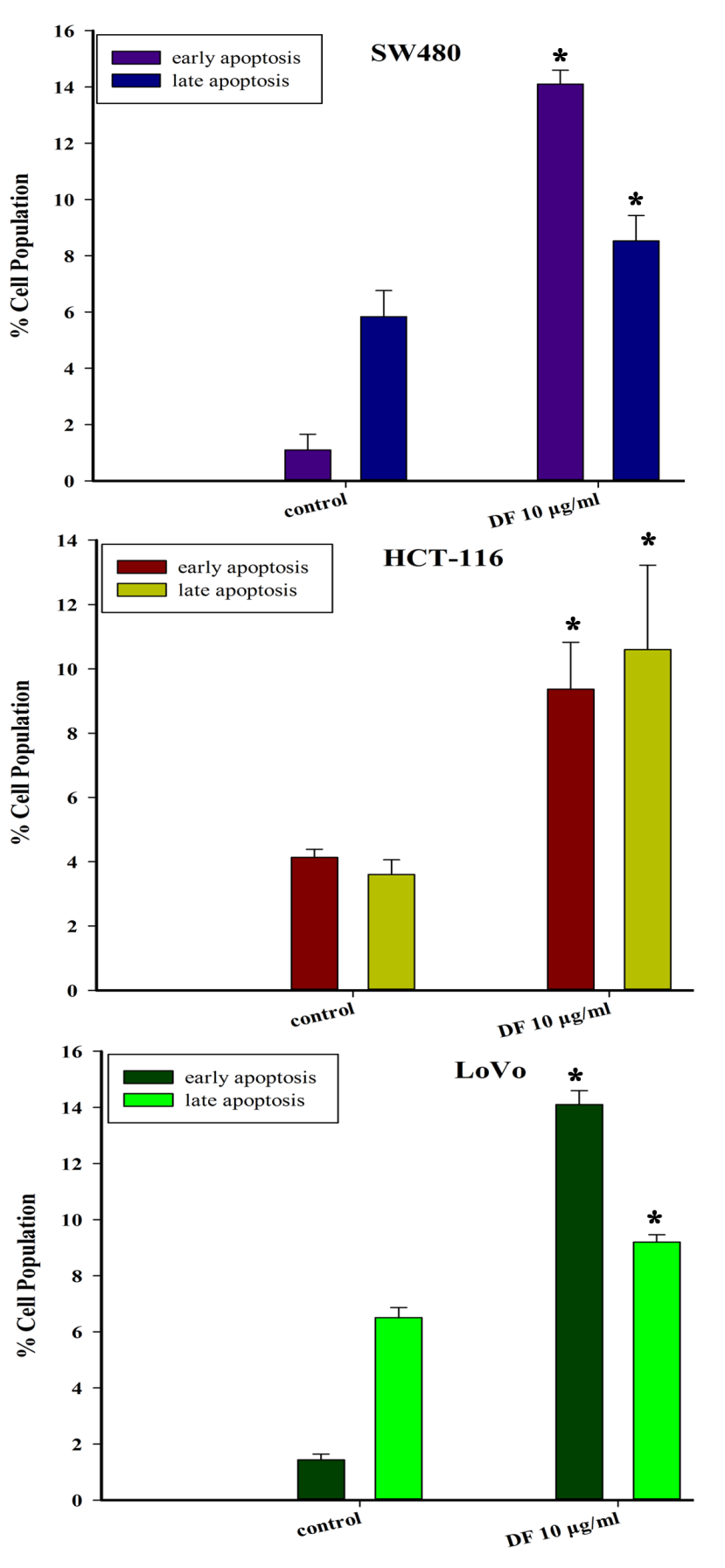


**Supplementary Figure 3 |** The effects of DF on the externalization of PS in three human colon cancer cell lines, SW480 cells, HCT-116 cells, LoVo cells. Cells were treated with DMSO (control) and DF (10 μg/ml) for 24 h. Results were presented as mean (n = 3) ± SD., *, P ≤ 0.05.
